# Real-world data of tirzepatide in obesity management: a multicenter study by the Italian Society of Obesity – Campania Region

**DOI:** 10.17179/excli2025-9067

**Published:** 2026-01-14

**Authors:** Luigi Barrea, Ludovica Verde, Martina Galasso, Renato Patrone, Lucia Digitale, Alessandro Limardi, Marcello Orio, Giovanni Ragozzino, Luigi Digitale, Vittorio Salvatore, Antonella Savoia, Silvia Savastano, Annamaria Colao, Giovanna Muscogiuri

**Affiliations:** 1 Dipartimento di Psicologia e Scienze della Salute, Università Telematica Pegaso, Naples, Italy; 2 Department of Public Health, University of Naples Federico II, Naples, Italy; 3 Division of Endocrinology, Department of Medicine, The University of Arizona College of Medicine, Tucson, AZ, USA; 4 Centro Italiano per la cura e il Benessere del Paziente con Obesità (C.I.B.O), Unità di Endocrinologia, Diabetologia e Andrologia, Dipartimento di Medicina Clinica e Chirurgia, Università degli Studi di Napoli Federico II, Naples, Italy; 5 Unit of Abdominal Oncology, Division of Hepatobiliary Surgical Oncology,Istituto Nazionale Tumori, Fondazione G. Pascale, IRCCS, Naples, Italy; 6 Ambulatorio di Obesiologia, Endocrinologia e Nutrizione Clinica - Centro Medico Polispecialistico DIGIMED, Naples, Italy; 7 U.O.S. Fasce Deboli e Cure Domiciliari DSB 33, Naples, Italy; 8 Centro Medico Specialistico Orio CMSO, Salerno, Italy; 9 Ambulatorio di Endocrinologia, Diabetologia e Nutrizione Clinica, Dipartimento di Scienze e Tecnologie Ambientali, Biologiche e Farmaceutiche (DiSTABiF), Università degli studi della Campania, Caserta, Italy; 10 UOC Medicina Interna, AOU San Giovanni di Dio e Ruggi D'Aragona, Salerno, Italy; 11 Ambulatorio di Endocrinologia, ASL Napoli 2 Nord, Naples, Italy; 12 Dipartimento di Medicina Clinica e Chirurgia, Unità di Endocrinologia, Diabetologia ed Andrologia, Università degli Studi di Napoli Federico II, Naples, Italy; 13 Cattedra Unesco "Educazione Alla Salute E Allo Sviluppo Sostenibile", Università degli Studi di Napoli Federico II, Naples, Italy

**Keywords:** obesity management, real-world study, tirzepatide, metabolic improvement, GIP/GLP-1 receptors agonists

## Abstract

Obesity is a growing public health concern, closely linked to metabolic and cardiovascular complications. Tirzepatide, a dual glucose-dependent insulinotropic polypeptide (GIP) and glucagon-like peptide-1 (GLP-1) receptor agonist, has shown substantial weight loss effects in clinical trials; however, real-world data, especially at lower doses, remain limited. This study aimed to evaluate the short-term effects of tirzepatide 2.5 mg and 5.0 mg on weight, metabolic parameters, and tolerability in adults with obesity in a real-world outpatient setting. This retrospective multicenter study included 70 adults with obesity but without type 2 diabetes, treated with tirzepatide between January and June 2025 in the Campania Region, Italy. Anthropometric and biochemical parameters were assessed at baseline. Follow-up data were collected at dose transitions: from 2.5 mg to 5.0 mg, and from 5.0 mg to 7.5 mg, allowing assessment of the effects of the 2.5 mg and 5.0 mg doses. Seventy participants were included (mean age 50.7 ± 10.2 years; 60 % female; BMI 37.5 ± 6.8 kg/m2). Treatment led to dose-dependent reductions in body weight, BMI, and waist circumference (p < 0.001 for all vs baseline and between doses). Significant improvements were observed in total cholesterol (p = 0.006 between doses), LDL cholesterol (p = 0.001), triglycerides (p < 0.001), prediabetes prevalence (p < 0.001 vs baseline; p = 0.002 between doses), fasting plasma glucose (p < 0.001 vs baseline; p < 0.001 between doses), insulin (p < 0.001 vs baseline; p < 0.001 between doses), HoMA-IR (p < 0.001 vs baseline; p < 0.001 between doses), AST (p = 0.012 vs baseline; p = 0.006 between doses), and ALT (p < 0.001 vs baseline; p = 0.007 between doses). Amylase increased significantly only at 5.0 mg (p = 0.016), while lipase remained unchanged. Renal function (eGFR) improved at both doses (p = 0.025 for 2.5 mg; p = 0.005 for 5.0 mg). Gastrointestinal adverse events were mild and similar between doses. In this real-world cohort, tirzepatide at 2.5 mg and 5.0 mg led to substantial improvements in weight and metabolic health, with good tolerability. These findings support its use in routine obesity care and justify further longitudinal research.

See also the graphical abstract(Fig. 1).

## Introduction

Obesity is a widespread and chronic condition with a relapsing course, and its prevalence continues to rise globally (Shah et al., 2025[22]; Verde et al., 2024[23]). In Italy, recent data from the Italian Barometer Obesity Report 2024 indicate that 11.8 % of adults are living with obesity, while an additional 34.6 % are overweight, resulting in nearly 46.3 % of the adult population being affected by excess weight (IBDO Foundation, 2024[14]). Although Italy reports lower obesity rates than the European average, the prevalence has increased by over 30 % in the past two decades, highlighting a growing public health concern (IBDO Foundation, 2024[14]).

Obesity is closely associated with several comorbidities, including type 2 diabetes (T2D), cardiovascular disease (CVD), hypertension, and various forms of cancer, all of which contribute to increased mortality and reduced life expectancy (Powell-Wiley et al., 2021[21]). In fact, obesity has been linked to a measurable decline in national life expectancy and places a significant burden on healthcare systems due to elevated medical costs and diminished productivity (Zhou et al., 2024[27]).

Clinical evidence suggests that a weight loss of at least 5 %-10 % from baseline is associated with meaningful improvements in metabolic and cardiovascular risk factors (Yurkow et al., 2023[26]). While lifestyle interventions, such as dietary modifications, increased physical activity, and behavioral therapy, represent the first line of treatment, maintaining long-term adherence remains a challenge (Allocca et al., 2025[2]; Barrea et al., 2025[7]). As a result, pharmacologic therapies have become an important adjunct to lifestyle interventions, particularly for individuals with a body mass index (BMI) ≥30.0 kg/m² or ≥27.0 kg/m² in the presence of obesity-related complications (Allocca et al., 2025[2]; Barrea et al., 2025[5][6]).

Tirzepatide is a once-weekly dual agonist of the glucose-dependent insulinotropic polypeptide (GIP) and glucagon-like peptide-1 (GLP-1) receptors (Jastreboff et al., 2022[15]). It received approval in the United States for obesity in November 2023 and in Europe in April 2024. In the phase 3 SURMOUNT clinical trials, tirzepatide showed significant weight loss effects in adults with obesity or overweight when compared to placebo. In the SURMOUNT-1 study, individuals with obesity but without T2D who were treated with 15 mg of tirzepatide experienced an average weight reduction of 22.5 % over 72 weeks, compared to 2.4 % in the placebo group (efficacy estimand) (Jastreboff et al., 2022[15]). In the SURMOUNT-3 trial, participants first underwent a 12-week intensive lifestyle intervention, followed by 72 weeks of tirzepatide treatment at the maximum tolerated dose (10 or 15 mg), resulting in an average total weight loss of 26.6 %. In contrast, those who continued with the placebo after the lifestyle phase lost only 3.8 % of their weight (Gibble et al., 2025[12]). The SURMOUNT-4 study revealed that individuals who remained on tirzepatide at the maximum tolerated dose following a 36-week lead-in period experienced an additional 6.7 % weight reduction over the next 52 weeks (Aronne et al., 2024[3]). Meanwhile, participants who switched to placebo during this phase regained an average of 14.8 % of their weight (efficacy estimand) (Aronne et al., 2024[3]).

Despite the robust efficacy observed in clinical trials, evidence on the effectiveness, tolerability, and safety of tirzepatide in real-world clinical settings-especially at early or lower doses-remains limited. Studying short treatment durations is clinically relevant, even if tirzepatide is not expected to be used only briefly for modest weight loss. The first weeks of therapy represent a critical initiation phase, during which patients start at the lowest dose (2.5 mg) before titration. Understanding outcomes in this phase helps set realistic expectations for both patients and clinicians. Moreover, tolerability issues, particularly gastrointestinal adverse events, often emerge early and can influence adherence and long-term continuation. Finally, documenting early real-world outcomes fills an important knowledge gap, as most clinical trials primarily report long-term results. Thus, the aim of the present study was to evaluate the short-term effects of tirzepatide 2.5 mg and 5.0 mg on weight changes and modifications in metabolic parameters in adults with obesity in a real-world outpatient setting and to assess its tolerability profile at these initial doses. 

## Materials and Methods

### Study design and population

This retrospective, multicenter study included adults with obesity and without T2D who were prescribed tirzepatide for weight management. Data were collected retrospectively from multiple outpatient clinics across Italy between January and June 2025. Patients with a diagnosis of T2D were excluded from the analysis.

### Data collection

Participating clinicians entered patient data into a standardized online form developed using Google Forms. The form was designed to collect anonymized, structured clinical information, including demographic characteristics (age, sex), lifestyle factors (smoking status, physical activity), comorbidities (prediabetes, hypertension, dyslipidemia, cardiovascular disease), anthropometric measurements (weight, height, and waist circumference), and details on tirzepatide treatment duration and dosage.

Biochemical parameters recorded at baseline included total cholesterol, HDL cholesterol, LDL cholesterol, triglycerides, amylase, lipase, aspartate aminotransferase (AST), alanine aminotransferase (ALT), fasting plasma glucose, and fasting insulin. The latter two were used to calculate the Homeostatic Model Assessment for Insulin Resistance (HoMA-IR), using the following formula (Matthews et al., 1985[19]):

*HoMA-IR = (fasting plasma glucose [mg/dL] × fasting insulin [mIU/mL]) / 405.*

Additionally, estimated glomerular filtration rate (eGFR) was collected. Follow-up data included updated anthropometric measures, biochemical parameters, and adverse event occurrence and were collected at visits corresponding to the transition from 2.5 mg to 5.0 mg and from 5.0 mg to 7.5 mg of tirzepatide in order to assess the effects of the 2.5 mg and 5.0 mg dosing regimens. The number of available observations for each parameter varied due to differences in clinical practice and data availability across sites. All data were entered by healthcare professionals at the respective clinical sites, based on medical records and patient-reported information obtained during routine outpatient visits.

### Statistical analysis

Descriptive statistics were used to summarize baseline characteristics. Variables with non-normal distributions were logarithmically transformed prior to analysis. Continuous variables were reported as means ± standard deviations (SD), while categorical variables were expressed as absolute frequencies and percentages. Comparisons from baseline and between tirzepatide 2.5 mg and 5.0 mg were performed using paired *t*-tests for continuous variables and chi-square tests for categorical variables. A two-tailed p value <0.05 was considered statistically significant. All analyses were conducted using SPSS Statistics software, version 22 (IBM Corp., Armonk, NY, USA).

## Results

A total of 70 participants were included in the analysis. The mean (±SD) age was 50.7 ± 10.2 years, and the majority were female (42 participants, 60.0 %). Regarding lifestyle characteristics, 12 participants (17.4 %) reported being current smokers, while 34 (49.3 %) reported engaging in regular physical activity. With respect to comorbidities, prediabetes was present in 29 participants (43.3 %), hypertension in 30 (47.6 %), dyslipidemia in 39 (57.4 %), and cardiovascular disease in 11 (15.7 %). The mean duration of treatment with tirzepatide at the 2.5-mg dose was 4.2 ± 0.7 weeks while the mean duration at the 5.0 mg dose was 13.1 ± 5.4 weeks (Table 1(Tab. 1)).

### Efficacy

Among the 70 participants, treatment with tirzepatide led to significant improvements in anthropometric and metabolic parameters (Table 2(Tab. 2)). 

Body weight decreased from 105.9 ± 23.6 kg at baseline to 100.9 ± 22.6 kg after treatment with tirzepatide 2.5 mg (p < 0.001), and further declined to 92.6 ± 22.2 kg with 5.0 mg (p < 0.001 *vs* baseline; p < 0.001 comparing 2.5 mg with 5.0 mg). Similarly, BMI decreased from 37.5 ± 6.8 kg/m² at baseline to 35.7 ± 6.5 kg/m² (p < 0.001), and to 32.7 ± 6.4 kg/m² (p < 0.001 *vs *baseline; p < 0.001 comparing 2.5 mg with 5.0 mg). The proportion of participants with a BMI ≥40.0 kg/m² decreased from 30.0 % at baseline to 24.3 % with 2.5 mg, and to 12.9 % with 5.0 mg. Waist circumference decreased significantly from 117.8 ± 16.1 cm to 113.1 ± 16.7 cm at 2.5 mg (p < 0.001), and to 104.8 ± 15.7 cm at 5.0 mg (p < 0.001 *vs* baseline; p < 0.001 comparing 2.5 mg with 5.0 mg). 

Improvements were observed in the lipid profile, with reductions in total cholesterol (from 201.3 ± 40.7 mg/dl to 189.2 ± 33.2 mg/dl at 2.5 mg, and 179.1 ± 28.3 mg/dl at 5.0 mg; p = 0.006 comparing 2.5 mg with 5.0 mg), LDL cholesterol (from 129.8 ± 31.0 to 115.3 ± 27.5 and 102.6 ± 21.7 mg/dl, respectively; p = 0.001 comparing 2.5 mg with 5.0 mg), and triglycerides (from 153.4 ± 51.4 to 139.8 ± 40.9 and 124.4 ± 34.8 mg/dl; p < 0.001 comparing 2.5 mg with 5.0 mg). HDL cholesterol increased modestly (from 47.4 ± 13.6 to 50.3 ± 13.4 and 52.2 ± 11.7 mg/dl; p = 0.059 comparing 2.5 mg with 5.0 mg).

A significant reduction in prediabetes prevalence was observed: from 48.6 % at baseline to 31.4 % with 2.5 mg (p < 0.001) and to 11.4 % with 5.0 mg (p = 0.002 comparing 2.5 mg with 5.0 mg). Fasting plasma glucose, insulin, and HoMA-IR all showed marked reductions: fasting plasma glucose declined from 103.6 ± 14.1 to 95.6 ± 14.4 and 90.2 ± 12.1 mg/dl (p < 0.001); insulin from 27.8 ± 11.8 to 20.8 ± 8.4 and 15.7 ± 6.8 mIU/ml (p < 0.001); and HoMA-IR from 7.3 ± 3.2 to 5.1 ± 2.1 and 3.6 ± 1.6 (p < 0.001), with all showing significant improvements comparing 2.5 mg with 5.0 mg (p < 0.001 for all).

**Figure 2:**
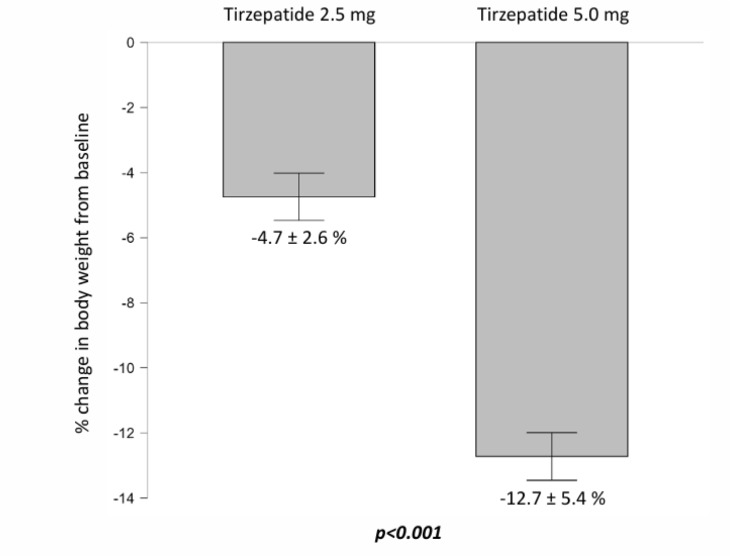
Percentage change in body weight from baseline after treatment with Tirzepatide 2.5 mg and Tirzepatide 5 mg

Treatment with tirzepatide was associated with a dose-dependent reduction in body weight. The mean percentage change in body weight was -4.7 ± 2.6 % with the 2.5-mg dose and -12.7 ± 5.4 % with the 5.0-mg dose (p < 0.001 *vs* baseline) (Table 3(Tab. 3); Figure 2(Fig. 2)). After treatment with 2.5 mg, 61.4 % of participants experienced less than 5 % weight loss, compared with only 2.9 % at 5.0 mg. Conversely, 97.1 % of participants receiving 5.0 mg achieved more than 5 % weight loss, compared with 38.6 % of those receiving 2.5 mg. Furthermore, 68.6 % of participants in the 5.0-mg group achieved more than 10 % weight loss, compared to only 4.3 % in the 2.5-mg group. A weight reduction greater than 15 % was observed in 27.2 % of participants treated with 5.0 mg, versus 1.4 % with 2.5 mg.

### Safety

**Figure 3:**
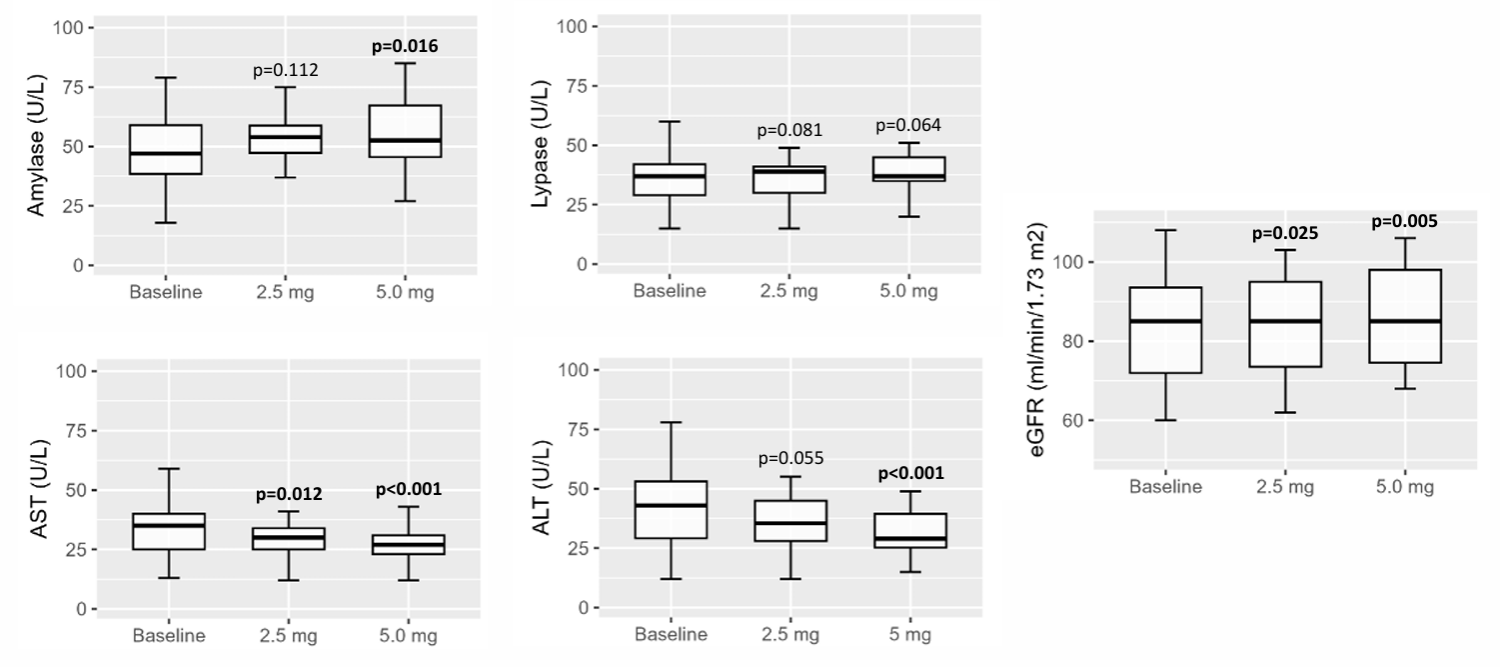
Changes in pancreatic, hepatic, and renal parameters from baseline after treatment with Tirzepatide at 2.5 mg and 5.0 mg. Improvements were observed in liver enzymes and renal function, while amylase increased only at the higher dose. A p-value in bold type denotes a significance difference (p < 0.05). *Abbreviations*: AST, aspartate transaminase; ALT, alanine transaminase; eGFR, estimated glomerular filtration rate

For amylase, a significant increase was observed at 5.0 mg compared to baseline (from 52.0 ± 15.8 to 56.5 ± 18.7 U/L; p = 0.016), while the change at 2.5 mg and the between-group comparison were none statistically significant (Table 4(Tab. 4); Figure 3(Fig. 3)). No significant changes were detected for lipase at either dose. Compared with baseline, AST levels decreased significantly with both 2.5 mg (p = 0.012) and 5.0 mg (p < 0.001), with a significant between-group difference (from 34.6 ± 11.5 to 31.1 ± 10.1 at 2.5 mg and 28.9 ± 9.5 U/L at 5.0 mg; p = 0.006). Similarly, ALT levels were significantly reduced at 5.0 mg (p < 0.001) compared to baseline, with a between-group difference also reaching significance (from 41.6 ± 15.7 to 36.0 ± 12.5 and 32.1 ± 10.3 U/L, respectively; p = 0.007).

Renal function, assessed by eGFR, improved significantly from baseline with both doses (from 83.2 ± 13.2 to 84.6 ± 12.0 ml/min/1.73 m² at 2.5 mg, p = 0.025, and to 85.6 ± 11.6 ml/min/1.73 m² at 5.0 mg, p = 0.005); however, the difference between tirzepatide 2.5 mg and 5 mg did not reach statistical significance, although a trend toward greater improvement with the higher dose was observed (Figure 3(Fig. 3)).

**Figure 4:**
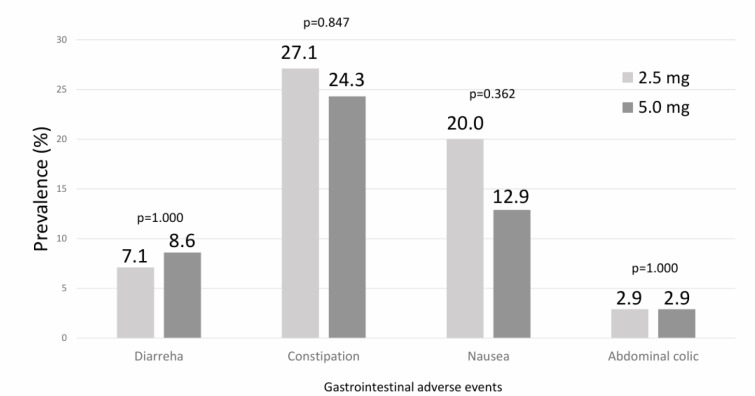
Gastrointestinal adverse events after treatment with Tirzepatide at 2.5 mg and 5.0 mg. Most events were mild and comparable between doses. A p-value in bold type denotes a significance difference (p < 0.05).

The only reported adverse events were gastrointestinal events at both dose levels of tirzepatide (Figure 4(Fig. 4)). The most frequently reported symptoms were constipation (27.1 % of participants at 2.5 mg and 24.3 % at 5.0 mg) and nausea (20.0 % at 2.5 mg and 12.9 % at 5.0 mg). Diarrhea was less common, occurring in 7.1 % of participants at 2.5 mg and 8.6 % at 5.0 mg. Abdominal colic was infrequent, reported in 2.9 % of participants in both treatment groups. All differences between doses were not statistically significant (Table 5(Tab. 5)).

## Discussion

In this real-world study, tirzepatide demonstrated substantial and dose-dependent benefits in managing obesity and its associated metabolic disturbances in individuals with obesity and without T2D. The observed improvements spanned anthropometric, metabolic, hepatic, and renal domains, confirming tirzepatide's multifaceted potential as a therapeutic agent beyond glycemic control (Liu et al., 2025[18]).

One of the most striking finding was the significant reduction in body weight, which was clearly dose-dependent. This was reflected not only in absolute weight reduction but also in clinically meaningful decreases in BMI and waist circumference, critical markers of obesity severity and central adiposity (Barazzoni et al., 2019[4]). Consequently, the proportion of individuals with severe obesity substantially declined with treatment escalation, showing tirzepatide's potential to shift patients from high-risk obesity categories into more manageable weight strata. Given the well-established relationship between obesity, particularly visceral fat accumulation, and cardiovascular risk, these anthropometric changes are likely to translate into meaningful clinical benefits over the longer term (Powell-Wiley et al., 2021[21]). In addition, supportive evidence from real-world studies conducted in other populations reinforces the generalizability of these findings (Adamidis et al., 2025[1]; Hankosky et al., 2025[13]). In a prospective observational study from Greece, adults with obesity treated with tirzepatide in routine clinical practice experienced rapid early reductions in body weight, waist circumference, and fat mass, with preservation of fat-free mass (Adamidis et al., 2025[1]). Consistently, a large U.S. retrospective analysis of adults without T2D initiating tirzepatide reported substantial weight loss at 6 months, even with slower dose escalation than in clinical trials (Hankosky et al., 2025[13]).

Metabolic improvements paralleled these anthropometric gains. There was a notable decrease in the prevalence of prediabetes, coupled with significant improvements in fasting plasma glucose, insulin levels, and HoMA-IR. From a clinical perspective, the observed reduction in prediabetes prevalence and improvement in insulin resistance reinforce tirzepatide's potential role in diabetes prevention, a critical objective in populations at high cardiometabolic risk, with important implications for long-term morbidity and healthcare burden (Galicia-Garcia et al., 2020[10]). This is further supported by the 3-year extension of the SURMOUNT-1 trial, which showed a markedly lower incidence of T2D in individuals with prediabetes treated with tirzepatide, compared to placebo (Jastreboff et al., 2025[16]).

The study also showed favorable modifications in lipid profiles, with reductions in total cholesterol, LDL cholesterol, and triglycerides, and a modest increase in HDL cholesterol. These changes are clinically relevant considering the high burden of dyslipidemia in the study population and the pivotal role of lipid abnormalities in cardiovascular morbidity and mortality (Mattiuzzi et al., 2020[20]). 

Liver enzymes, particularly AST and ALT, decreased significantly during treatment, suggesting potential hepatic benefits. Given the high prevalence of multi-organ dysfunction in individuals with obesity and metabolic disease, the concurrent improvements in hepatic enzymes and renal function observed in this real-world setting are clinically relevant and suggest a potential role for tirzepatide in integrated organ protection strategies beyond weight loss and glycemic control (Latif and Ahsan, 2024[17]). Improvements in hepatic enzyme levels may indicate reductions in hepatic inflammation or steatosis, which, if sustained, could translate into improved liver health and a reduced risk of progression toward more advanced forms of steatotic liver disease, such as metabolic dysfunction-associated steatohepatitis (MASH) in the presence of the required risk factors, or even cirrhosis (Basil et al., 2024[8]). 

Renal function, assessed by eGFR, showed a trend toward improvement, which is encouraging in a population at increased risk for chronic kidney disease due to metabolic comorbidities. The observed improvements from baseline with both doses, suggests that tirzepatide might exert a protective or stabilizing effect on renal function, a hypothesis supported by emerging data on incretin-based therapies in diabetic kidney disease (Caruso and Giorgino, 2024[9]; Verde et al., 2023[24]).

Importantly, the low incidence and comparable rates of gastrointestinal adverse events between doses suggest that appropriate patient counseling and gradual titration may support long-term adherence in routine clinical practice. The absence of clinically relevant pancreatic adverse events provides additional reassurance regarding the pancreatic safety of tirzepatide in a real-world setting. Mild increases in amylase were observed at the higher dose, whereas changes in lipase were not significant.

The real-world nature of this study provides important complementary evidence to randomized controlled trials (RCTs), such as those in the SURMOUNT studies (Aronne et al., 2024[3]; Garvey et al., 2023[11]; Jastreboff et al., 2022[15]; Wadden et al., 2023[25]) which have already demonstrated the efficacy and safety of tirzepatide under controlled conditions. However, it should be emphasized that SURMOUNT-1, -3, and -4 involved much longer treatment durations at higher doses, with carefully selected populations and controlled follow-up schedules. Therefore, while comparisons can provide context, the differences in population characteristics, dosing regimens, and duration of treatment must be acknowledged to avoid overstating equivalence. Real-world data capture the complexity and heterogeneity of clinical practice, including patients with multiple comorbidities, varied adherence patterns, and less rigid treatment protocols. Despite these challenges, the consistent and robust benefits observed in this cohort reinforce tirzepatide's translational potential (Aronne et al., 2024[3]; Garvey et al., 2023[11]; Jastreboff et al., 2022[15]; Wadden et al., 2023[25]).

Compared to RCTs findings the magnitude of weight loss and metabolic improvement in this study is comparable, supporting the external validity of clinical trial results (Aronne et al., 2024[3]; Garvey et al., 2023[11]; Jastreboff et al., 2022[15]; Wadden et al., 2023[25]). Importantly, the real-world setting underscores tirzepatide's effectiveness in everyday clinical practice, where patients often present with multiple concurrent health issues and variable lifestyle factors. The inclusion of participants with a substantial burden of hypertension, dyslipidemia, CVD, and prediabetes highlights the drug's utility in managing complex patient profiles frequently encountered outside of trials. 

Furthermore, the study sheds light on dose-dependent effects in a pragmatic context. While RCTs provide controlled dose escalation schedules (Aronne et al., 2024[3]; Garvey et al., 2023[11]; Jastreboff et al., 2022[15]; Wadden et al., 2023[25]), real-world data suggest that titration to the 5.0 mg dose yields superior outcomes without compromising tolerability. This supports clinical decision-making around personalized dosing to maximize benefit-risk ratios in routine care.

Several limitations should be acknowledged when interpreting these findings. The retrospective real-world design is inherently subject to selection bias, missing data, and the absence of a randomized comparator, and the unequal duration of exposure at the two dose levels limits any formal assessment of dose effects independent of time-on-treatment. Nevertheless, these features reflect routine clinical practice and represent a strength in terms of external validity, capturing the heterogeneity and complexity of patients typically excluded from randomized trials. The consistency of early improvements across multiple cardiometabolic domains, together with their alignment with evidence from RCTs and international real-world studies, supports the robustness and clinical relevance of the present findings despite the relatively short observation period (Aronne et al., 2024[3]; Garvey et al., 2023[11]; Jastreboff et al., 2022[15]; Wadden et al., 2023[25]).

In conclusion, tirzepatide treatment in a real-world cohort with high cardiometabolic risk resulted in robust, dose-dependent improvements in weight, metabolic control, lipid profile, liver function, and potentially renal function. These findings, however, reflect early treatment effects observed at initial doses, rather than long-term or durable outcomes. Nevertheless, the comprehensive benefits, coupled with an acceptable safety profile, support tirzepatide's use as an effective therapeutic option in the management of obesity and its associated metabolic complications beyond controlled clinical trial settings. Future longitudinal studies will be essential to confirm the durability of these early benefits and to clarify their impact on hard clinical outcomes, such as cardiovascular events and progression of diabetes and liver disease (Barrea et al., 2025[6]).

## Notes

Luigi Barrea, Ludovica Verde, and Martina Galasso contributed equally as first author.

## Declaration

### Acknowledgments

The authors wish to thank Ermenegilda Pagano, Maria Grazia Santaniello, Patrizia Colicchio, and all members of SIO Campania for their valuable support and contribution. 

### Author contributions

Design: LB and GM; Data collection: RP, LD, AL, MO, RG, LD, VS and AS; Analysis: LB and LV; Writing manuscript: LB, GM, LV and MG; Supervision: SS and AC.

### Competing interests

The authors have no competing interests to declare that are relevant to the content of this article.

### Funding

The authors did not receive support from any organization for the submitted work.

### Data availability statement

The data that support the findings of this study are available on request from the corresponding author. The data are not publicly available due to privacy or ethical restrictions.

### Using artificial intelligence (AI) 

We have not used any artificial intelligence (AI)-assisted technologies in the production of submitted work.

## Figures and Tables

**Table 1:**
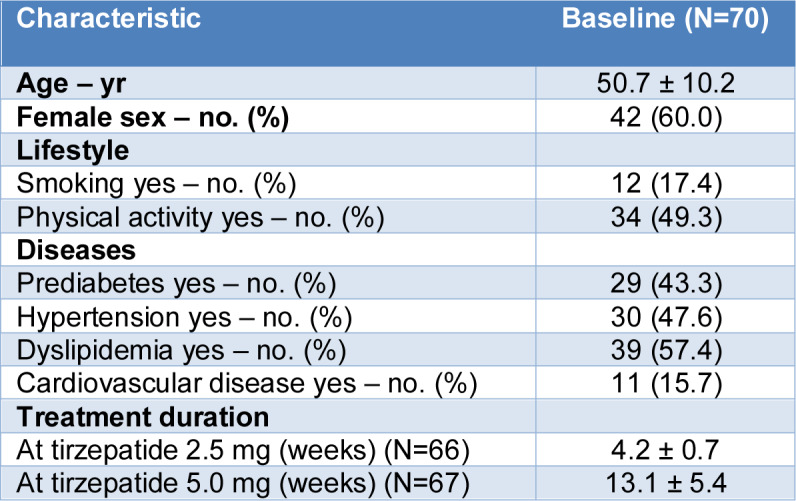
Baseline characteristics of the study population (N = 70)

**Table 2:**
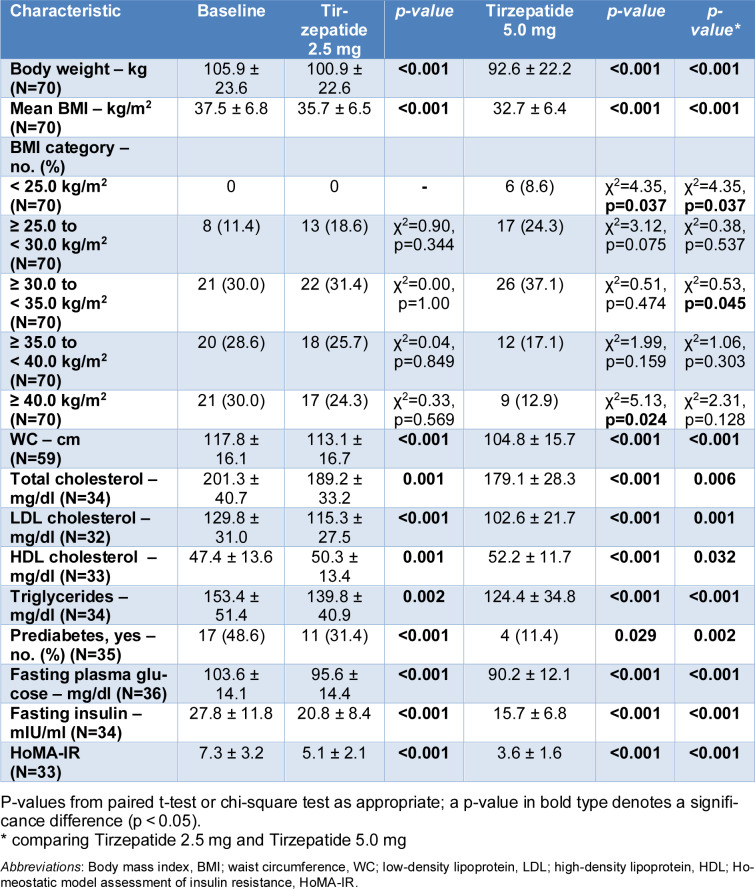
Changes in anthropometric and laboratory parameters from baseline to Tirzepatide 2.5 mg and 5.0 mg

**Table 3:**
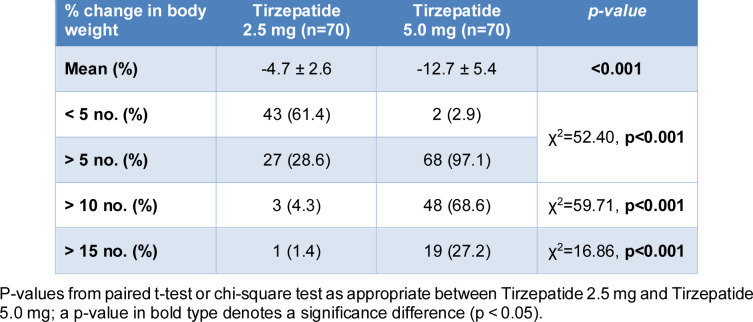
Percentage change in body weight after treatment with tirzepatide 2.5 mg and 5 mg

**Table 4:**
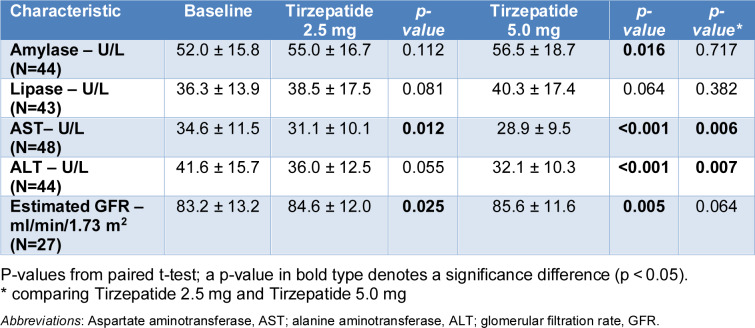
Changes in laboratory parameters from baseline to Tirzepatide 2.5 mg and 5.0 mg

**Table 5:**
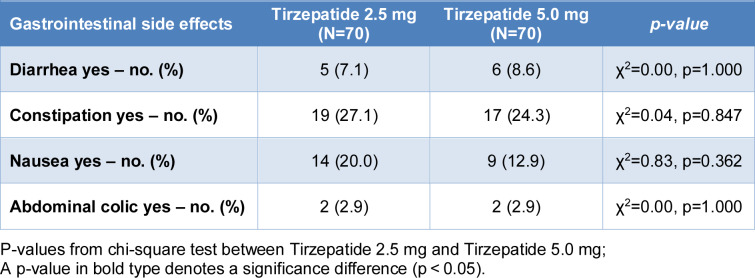
Gastrointestinal adverse events in participants treated with Tirzepatide 2.5 mg and 5 mg

**Figure 1:**
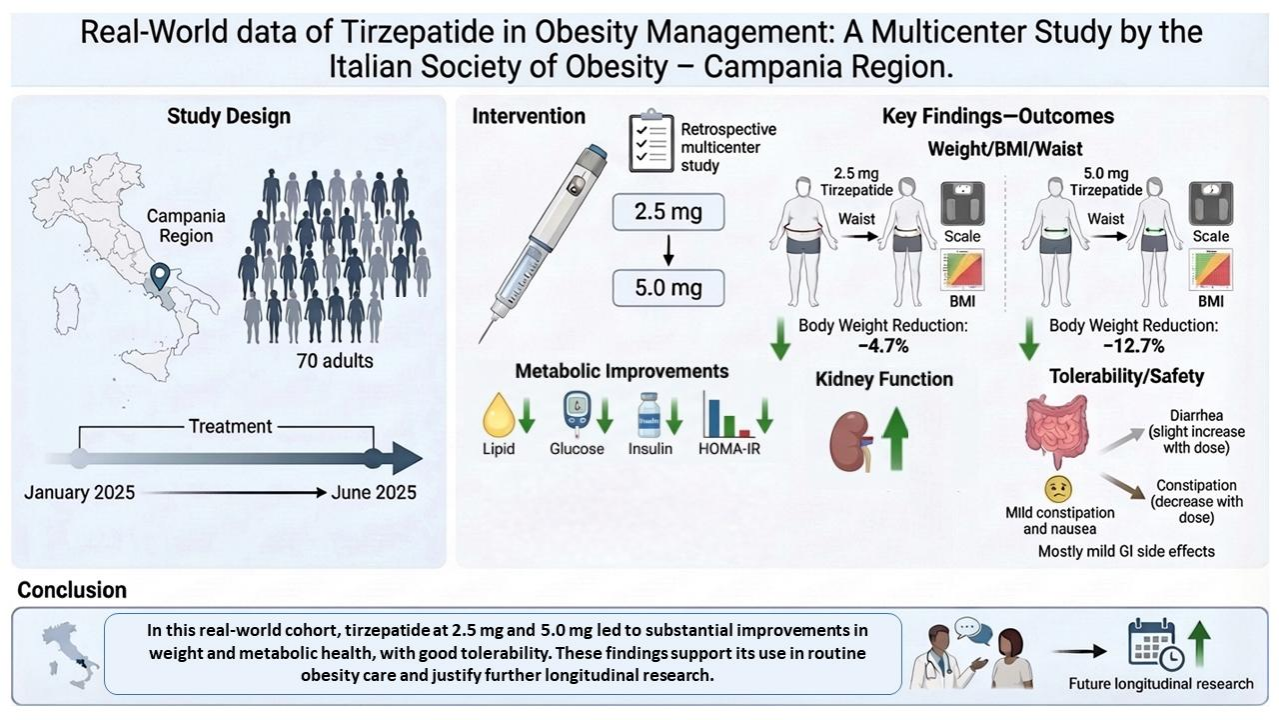
Graphical abstract

## References

[1] Adamidis N, Desalermos A, Papadopoulou N, Adamidi S, Koutrakos T, Kyventidou M (2025). Short-Term Effects of Tirzepatide in Obese Adults: A Real-World Prospective Study. Cureus.

[2] Allocca S, Monda A, Messina A, Casillo M, Sapuppo W, Monda V (2025). Endocrine and Metabolic Mechanisms Linking Obesity to Type 2 Diabetes: Implications for Targeted Therapy. Healthcare (Basel).

[3] Aronne LJ, Sattar N, Horn DB, Bays HE, Wharton S, Lin WY (2024). Continued Treatment With Tirzepatide for Maintenance of Weight Reduction in Adults With Obesity: The SURMOUNT-4 Randomized Clinical Trial. JAMA.

[4] Barazzoni R, Gortan Cappellari G, Semolic A, Ius M, Zanetti M, Gabrielli A (2019). Central adiposity markers, plasma lipid profile and cardiometabolic risk prediction in overweight-obese individuals. Clin Nutr.

[5] Barrea L, Annunziata G, Verde L, Galasso M, Savastano S, Colao A (2025). A Multidisciplinary Perspective on Semaglutide Treatment and Medical Nutrition Therapy in Obesity Management. Curr Obes Rep.

[6] Barrea L, Boschetti M, Gangitano E, Guglielmi V, Verde L, Muscogiuri G (2025). Long-Term Efficacy and Safety of Nutritional and Pharmacological Strategies for Obesity. Curr Obes Rep.

[7] Barrea L, Verde L, Colao A, Mandarino LJ, Muscogiuri G (2025). Medical nutrition therapy for the management of type 2 diabetes mellitus. Nat Rev Endocrinol.

[8] Basil B, Myke-Mbata BK, Eze OE, Akubue AU (2024). From adiposity to steatosis: metabolic dysfunction-associated steatotic liver disease, a hepatic expression of metabolic syndrome - current insights and future directions. Clin Diabetes Endocrinol.

[9] Caruso I, Giorgino F (2024). Renal effects of GLP-1 receptor agonists and tirzepatide in individuals with type 2 diabetes: seeds of a promising future. Endocrine.

[10] Galicía-Garcia U, Benito-Vicente A, Jebari S, Larrea-Sebal A, Siddiqi H, Uribe KB (2020). Pathophysiology of Type 2 Diabetes Mellitus. Int J Mol Sci.

[11] Garvey WT, Frias JP, Jastreboff AM, le Roux CW, Sattar N, Aizenberg D (2023). Tirzepatide once weekly for the treatment of obesity in people with type 2 diabetes (SURMOUNT-2): a double-blind, randomised, multicentre, placebo-controlled, phase 3 trial. Lancet.

[12] Gibble TH, Cao D, Forrester T, Fraseur Brumm J, Chao AM (2025). Tirzepatide and health-related quality of life in adults with obesity or overweight: Results from the SURMOUNT-3 phase 3 randomized trial. Diabetes Obes Metab.

[13] Hankosky ER, Desai K, Chinthammit C, Grabner M, Stockbower G, He X (2025). Real-world use and effectiveness of tirzepatide among people without evidence of type 2 diabetes in the United States. Diabetes Metab.

[14] Novelli G, Busetto L, Corsaro L, IBDO Foundation (2024). Obesity Monitor 2024 “Obesità: la pandemia del futuro”. A cura: IBDO Foundation;Istat, CREA SANITÀ, CORESEARCH;BHAVE.

[15] Jastreboff AM, Aronne LJ, Ahmad NN, Wharton S, Connery L, Alves B (2022). Tirzepatide Once Weekly for the Treatment of Obesity. N Engl J Med.

[16] Jastreboff AM, le Roux CW, Stefanski A, Aronne LJ, Halpern B, Wharton S (2025). Tirzepatide for Obesity Treatment and Diabetes Prevention. N Engl J Med.

[17] Latif S, Ahsan T (2024). Prevalence of Metabolic Dysfunction-associated Steatotic Liver Disease (MASLD) in Persons with Obesity and Type 2 Diabetes Mellitus: A Cross-sectional Study. Euroasian J Hepatogastroenterol.

[18] Liu L, Shi H, Xie M, Sun Y, Nahata MC (2025). The Efficacy and Safety of Tirzepatide in Patients with Diabetes and/or Obesity: Systematic Review and Meta-Analysis of Randomized Clinical Trials. Pharmaceuticals (Basel).

[19] Matthews DR, Hosker JP, Rudenski AS, Naylor BA, Treacher DF, Turner RC (1985). Homeostasis model assessment: insulin resistance and beta-cell function from fasting plasma glucose and insulin concentrations in man. Diabetologia.

[20] Mattiuzzi C, Sanchis-Gomar F, Lippi G (2020). Worldwide burden of LDL cholesterol: Implications in cardiovascular disease. Nutr Metab Cardiovasc Dis.

[21] Powell-Wiley TM, Poirier P, Burke LE, Despres JP, Gordon-Larsen P, Lavie CJ (2021). Obesity and Cardiovascular Disease: A Scientific Statement From the American Heart Association. Circulation.

[22] Shah A, Davarci O, Chaftari P, Avenatti E (2025). Obesity as a Disease: A Primer on Clinical and Physiological Insights. Methodist Debakey Cardiovasc J.

[23] Verde L, Barrea L, Bowman-Busato J, Yumuk VD, Colao A, Muscogiuri G (2024). Obesogenic environments as major determinants of a disease: It is time to re-shape our cities. Diabetes Metab Res Rev.

[24] Verde L, Luca S, Cernea S, Sulu C, Yumuk VD, Jenssen TG (2023). The Fat Kidney. Curr Obes Rep.

[25] Wadden TA, Chao AM, Machineni S, Kushner R, Ard J, Srivastava G (2023). Tirzepatide after intensive lifestyle intervention in adults with overweight or obesity: the SURMOUNT-3 phase 3 trial. Nat Med.

[26] Yurkow S, Ivezaj V, Grilo CM (2023). Improvements in cardiovascular disease risk factors associated with modest weight loss following treatment in patients with binge-eating disorder and obesity. Int J Eat Disord.

[27] Zhou XD, Chen QF, Yang W, Zuluaga M, Targher G, Byrne CD (2024). Burden of disease attributable to high body mass index: an analysis of data from the Global Burden of Disease Study 2021. EClinicalMedicine.

